# Preliminary Study on Pulse Wave Changes in Patients with Inflammatory Arthropathies Treated with bDMARDs

**DOI:** 10.3390/jcm13092684

**Published:** 2024-05-02

**Authors:** Michela Gasparotto, Giuliano Di Pierro, Barbara Toffoli, Andrea Grillo, Marco Bressan, Marco Fiorentin, Lorenzo Di Luozzo, Fabio Fischetti, Margherita Zen, Bruno Fabris, Stella Bernardi, Paola Tomietto

**Affiliations:** 1 UCO Medicina Clinica, ASUGI, Cattinara Teaching Hospital, Strada di Fiume 447, 34149 Trieste, Italy; michela.gasparotto@asugi.sanita.fvg.it (M.G.); andrea.grillo@units.it (A.G.); f.fischetti@fmc.units.it (F.F.) b.fabris@fmc.units.it (B.F.); paola.tomietto@asugi.sanita.fvg.it (P.T.); 2 Department of Medicine, Padua University Hospital, University of Padua, 35122 Padova, Italy; margherita.zen@unipd.it; 3 Department of Medical Surgical and Health Sciences, University of Trieste, Cattinara Teaching Hospital, Strada di Fiume 447, 34149 Trieste, Italy; giudip2695@gmail.com (G.D.P.); btoffoli@units.it (B.T.); marcobrsn@gmail.com (M.B.); betandof@gmail.com (M.F.); dott.diluozzo@gmail.com (L.D.L.)

**Keywords:** arterial tonometry, aortic stiffness, cardiovascular risk, arthritis, systemic inflammation

## Abstract

**Background:** Patients with inflammatory arthropathies exhibit an increased cardiovascular disease (CVD) risk as compared to the general population, which is not fully quantified by the conventional CVD risk scores. Biotechnological disease-modifying drugs (bDMARDs) have proved beneficial to reduce the overall CVD risk in these patients, although CVD remains a major cause of increased mortality. Since it has been shown that pulse wave parameters and in particular carotid–femoral pulse wave velocity (cfPWV) are predictors of CVD risk, the aim of this study was to evaluate their changes in patients with inflammatory arthropathies before and after bDMARD therapy. **Methods:** Pulse wave parameters were evaluated with applanation tonometry in patients with ankylosing spondylitis (AS), psoriatic arthritis (PsA), and rheumatoid arthritis (RA), before and after two years of bDMARD therapy. **Results:** At baseline, cfPWV was significantly associated with age (*p* < 0.001) and, among pulse wave parameters, the subendocardial viability ratio was negatively associated with C-reactive protein (CRP) (*p* = 0.04) and the HAQ-disability index (*p* = 0.03). At baseline, PsA patients showed a higher percentage of male subjects, higher CRP, and the highest cfPWV values (*p* = 0.048). After two years, pulse wave parameters improved in the AS and RA groups, but not in the PsA group. **Conclusions:** Our data confirm that pulse wave parameters are potentially reversible after bDMARD therapy, as they improved in AS and RA patients. In PsA patients, there were no changes, which may be due to the higher percentage of male subjects and higher baseline cfPWV values.

## 1. Introduction

Patients with systemic inflammatory rheumatic diseases have an increased risk of cardiovascular events as compared to the general population [1], and cardiovascular disease (CVD) is responsible for their increased morbidity and mortality [2]. This is due to the fact that the systemic inflammatory state that characterizes these conditions leads to an accelerated development and progression of atherosclerosis, which is an inflammatory disease of the vessels, and the leading cause of CVD [3,4]. In addition, this systemic inflammatory state promotes the stiffening of the vessels, a process otherwise known as arteriosclerosis [5]. Therefore, it is current opinion that traditional and non-traditional risk factors for CVD, such as the low-grade inflammatory state (i.e., disease activity) and the use of prolonged steroid-based therapies, contribute to the higher risk of CVD in patients with systemic inflammatory rheumatic diseases [6]. In line with this, the European Alliance of Associations for Rheumatology (EULAR) has recently published specific recommendations for the careful management of both traditional and disease-related CVD risk factors in these patients [7].

Among all the systemic rheumatic diseases, inflammatory arthropathies have the most significant epidemiological impact and they have been the most studied for CVD risk assessment [8,9]. Unfortunately, the commonly used algorithms for CVD risk stratification do not adequately apply to patients with inflammatory arthropathies as they underestimate their real CVD risk in most of the cases [10,11]. It has been recently shown that the analysis of arterial pulse wave and the measurement of pulse wave velocity (PWV), as assessed by applanation tonometry, allow to obtain a more comprehensive evaluation of vascular homeostasis and CVD risk [12]. Applanation tonometry is a non-invasive technique whereby it is possible to evaluate several pulse wave parameters including carotid–femoral (cf)PWV [12], which is the gold-standard way to measure aortic stiffness as well as an independent predictor of CVD risk [13,14]. The other main pulse wave parameters include the following: central systolic (SBPc) and diastolic (DBPc) blood pressure, augmentation index (AIx), subendocardial viability ratio (SEVR), left ventricular ejection time (LVET) and systolic slope (SysS). In detail, SBPc and DBPc correspond to the blood pressure level inside the heart and the aorta [15]. AIx represents the intensity of the pulse wave reflection, and it is a surrogate marker of both aortic stiffness and left ventricular systolic loading [16,17]. SEVR is an indicator of cardiac perfusion and coronary reserve [18]. LVET depends on left ventricular systolic function, heart rate, and vascular peripheral resistances [19]. SysS reflects the presence of an underlying vascular stenosis or obstruction.

Based on these premises, the aim of our study was to evaluate pulse wave parameters and cfPWV with the use of arterial tonometry in a cohort of patients suffering from inflammatory arthropathies before and after treatment with biotechnological disease-modifying drugs (bDMARDs).

## 2. Materials and Methods

### 2.1. Study Design

This is an observational prospective study including adult patients (>18 years), who were affected with rheumatoid arthritis (RA) or seronegative spondyloarthropathies (ankylosing spondylitis (AS) or psoriatic arthritis (PsA)), and who were prescribed therapy with a bDMARD according to the disease-specific international recommendations [20,21,22] (i.e., moderate/severe disease activity, inefficacy of cDMARDs and, in case of AS, bone oedema on MRI and/or high CRP). The bDMARDs considered were anti-tumor necrosis factor-α (anti-TNF-α), anti-interleukin (IL)12/23, anti-IL17A, CD80/86 inhibitor, and an anti-IL6 receptor.

Patients were consecutively selected at the Rheumatology Unit of Cattinara Teaching Hospital (UCO Medicina Clinica, ASUGI) between January 2019 and December 2021. Patients were included after providing informed consent to participate in this study. Then, they were evaluated at the beginning of therapy (at recruitment) and after 24 months. At each medical visit, patients underwent full clinical assessment, venous blood sampling for general biochemistries and cytokine analysis, as well as arterial tonometry.

### 2.2. Clinical Assessment

For every subject we collected full patient history (and CVD risk evaluation), anthropometric parameters and clinimetric indices. The clinimetric indices considered for this study were the 68 tender joint count (68-TJC), the 66 swollen joint count (66-SJC), the patient global assessment (PGA), and the Health Assessment Questionnaire Disability Index (HAQ-DI). In patients with AS and PsA, the Bath ankylosing spondylitis disease activity index (BASDAI) was also calculated.

### 2.3. General Biochemistries and Cytokine Analysis

General biochemistries, which were measured by autoanalyzer, included the following: C-reactive protein (CRP) level, erythrocyte sedimentation rate (ESR), and lipid profile, i.e., total cholesterol (TC), high-density lipoprotein cholesterol (HDL-C), and triglycerides (TG). The cytokines that were analyzed included osteoprotegerin (OPG) and the receptor activator of nuclear factor-kB ligand (RANKL). OPG was measured with the R&D #DY805 ELISA kit and RANKL was measured with the R&D #DY626 ELISA kit (R&D System, Minneapolis, MN, USA). Each ELISA kit was evaluated for intra-assay reproducibility by running 3 positive control samples (containing high, medium, and low concentration of the specific marker) in duplicate (coefficient of variation <10%). For the inter-assay reproducibility, 3 control samples of known concentration were tested in duplicate in separate plates and on different days (coefficient of variation <15%).

### 2.4. Arterial Tonometry

Arterial tonometry was performed by a trained operator using the PulsePen^®^ (DiaTecne S.r.l., Milano, Italy). Each patient laid in supine position. Peripheral blood pressure was measured using a digital sphygmomanometer (OMRON M6 COMFORT HEM-7321-E, OMRON Healthcare Europe B.V., Hoofddorp, The Netherlands). Pressure waveform calibration was based on the calculation of the mean arterial pressure ((diastolic arterial pressure + peripheral pulse pressure)/3). To measure cfPWV, i.e., the speed at which the pulse wave runs through the arterial system, we employed the sequential ECG-gated carotid and femoral artery recording method, whereby the tonometer is applied first at the patient’s neck and then at his groin. The sampling rate was 1 kHz and the recording time was 10 cardiac cycles long. To calculate the cfPWV, we measured the distance (in millimeters) between the carotid and femoral recording sites, which was multiplied by 0.8 according to current recommendations [17]. These measurements were performed twice at the carotid and femoral arteries. If there was a difference lower than 1 m/s between the two cfPWV recordings, the one with the higher quality index (QI) was considered. If the difference was greater than 1 m/s, a third measurement was performed and the one with the highest QI was chosen. The remaining pulse wave parameters were obtained from the carotid pulse wave analysis. These parameters included SBPc and DBPc, AIx, SEVR, LVET and SysS. Tonometric data were processed by the software WPulsePen 2.3.2 (WPP001-ETT—2.3.1; 2013–2019 DiaTecne s.r.l, Milan, Italy).

### 2.5. Statistical Analysis

Statistical analysis was performed using the software “R” (version 4.0.3; 2020 The R Foundation for Statistical Computing) and GraphPad Prism (version 8.0.2). A *p* value < 0.05 was considered for statistical significance. Shapiro–Wilk test was applied to quantitative variables to check for distribution normality. Quantitative variables were reported as median with interquartile range (IQR); qualitative variables were reported as absolute frequencies and percentages. Univariate correlations were measured with the Pearson or the Spearman test based on data distribution. For continuous variables, two group comparisons were performed with the *t*-test or the Wilcoxon test, while multiple comparisons were performed with the one-way ANOVA or the Kruskal–Wallis test followed by Dunn’s test, based on data distribution. Multivariate linear regression was used to explore the effect of patient variables on pulse wave parameters. Results were reported in terms of beta regression coefficient with 95% confidence interval. For longitudinal analysis within the same group, we used the Wilcoxon test for matched pairs.

## 3. Results

### 3.1. General Characteristics of the Population

We enrolled 36 adult patients affected with inflammatory arthopathies, including 30 women (83.3%) and 6 men (16.7%). A total of 16 (44.4%) patients suffered from AS, 10 (27.8%) suffered from PsA and 10 (27.8%) suffered from RA. Baseline patient characteristics are reported in Table 1.

The median age of the cohort was 60 (54–74) years, with AS being the youngest group and RA being the oldest group, *p* = 0.015 (AS vs. RA *p* = 0.011). The overall median disease duration was 2 (0.75–4.5) years. Groups differed in terms of sex, as male patients represented 12.5% of the AS group, 40% of the PsA group, and 0% of the RA group (*p* = 0.047). The majority of patients were taking an anti-TNF-α (66.7%) or an anti-IL17A (16.7%) therapy, while only a minority were prescribed an anti-IL6 receptor, a CD80/86 inhibitor, or an anti-IL12/23 drug.

With respect to CVD risk factors, there were no differences in terms of smoking history, BMI, diabetes mellitus, hypertension, lipid profile and statin lipid-lowering drugs. As for the history of diabetes mellitus, in the AS group there were two patients with type 1 DM that were treated with insulin, whose glycated hemoglobin levels were 5.7% and 6.7%. In the PsA group there were two patients with type 2 DM that were treated with metformin, whose glycated hemoglobin levels were 5.6% and 6.5%.

Otherwise, multi-group comparison showed that PsA patients had the highest PGA score (*p* = 0.038, PsA vs. RA *p* = 0.038) and the highest cfPWV (*p* = 0.048, AS vs. PsA *p* = 0.075).

### 3.2. Associations between Patient Characteristics and Pulse Wave Parameters

The associations between patient characteristics and pulse wave parameters are reported in Table 2. Consistent with the data reported in Table 1, cfPWV baseline values significantly differed between AS, PsA and RA (*p* = 0.048), as in PsA patients cfPWV was 9.9 m/s (IQR 8.4–11.60) as compared to 9.6 m/s (8.3–10.5) in RA and 7.9 m/s (7.5–8.7) in AS patients (PsA vs. AS, *p* = 0.075). In addition, there was a positive correlation between age and cfPWV (rho 0.54, *p* = 0.001), age and LVET (rho 0.38, *p* = 0.02) as well as age and SBPc (rho 0.47, *p* = 0.01). TC and LDL values were negatively correlated with the SysS (rho −0.52, *p* = 0.01, and rho −0.50, *p* = 0.02, respectively) while HDL values were positively correlated with SBPc and DBPc.

When looking at the inflammatory state, disease activity, and disability, we found that CRP values and HAQ-DI score were negatively correlated with SEVR (rho −0.34, *p* = 0.04 and rho −0.38, *p* = 0.03, respectively), while the number of swollen joints was negatively correlated with LVET (rho −0.34, *p* = 0.05).

As for cytokine assessment, OPG levels were positively associated with cfPWV (rho 0.42, *p* = 0.01) and SBPc (rho 0.35, *p* = 0.04), while RANKL did not show any significant correlation.

Then, to explore the effect of patient variables on pulse wave parameters and to identify independent associations, we performed multivariate linear regression analyses. Only variables that were associated with a *p*-value < 0.10 with pulse wave parameters (Table 2) were considered for multivariate linear regression. Although there is a lack of consensus on the appropriate sample size for multivariate linear regression, we considered that only 3 predictive/independent variables could be tested in every model as our population consisted of 36 patients. Age and sex were always included. So, we tested the relationship of age, sex, and OPG (predictive variables) with cfPWV (dependent variable); the relationship of age, sex and disease group with cfPWV; the relationship of age, sex, and OPG with SBPc; the relationship of age, sex, and CRP with SEVR; and the relationship of age, sex, and HAQ-DI with SEVR.

In the end, these analyses showed that age was independently associated with cfPWV (*p* = 0.03) as well as cSBP (*p* = 0.035), while HAQ-DI was independently associated with SEVR (*p* = 0.016), as shown in Table 3.

### 3.3. Longitudinal Analysis of Pulse Wave Parameters and Disease Activity Indices in AS, PsA, and RA Patients

**Figure 1:**
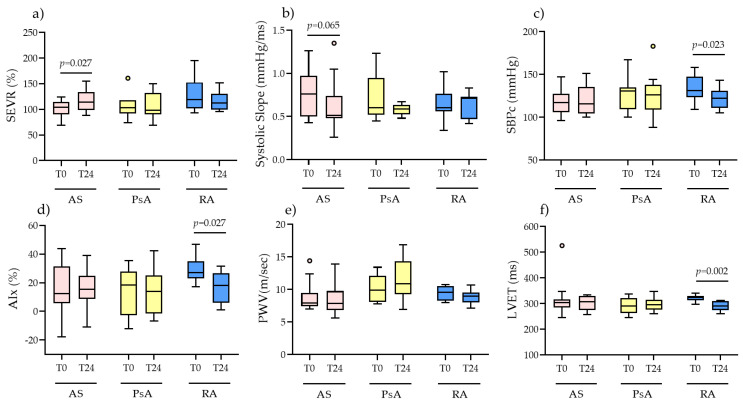
Longitudinal analysis of pulse wave parameters and aortic stiffness. Changes in (**a**) subendocardial viability ratio (SEVR); (**b**) systolic slope (SysS); (**c**) central systolic blood pressure (SBPc); (**d**) augmentation index (AIx); (**e**) carotid–femoral pulse wave velocity (cfPWV); (**f**) left ventricular ejection time (LVET). Results were reported as median + IQR. Circles are outliers. Comparisons were performed with Wilcoxon matched pairs signed rank test.

We prospectively analyzed the changes in pulse wave parameters and clinimetric indices from T0 to T24 in the three different inflammatory arthropathies to assess the impact of bDMARD therapy on pulse wave, aortic stiffness, and disease activity. With respect to pulse wave parameters and aortic stiffness, AS patients exhibited a significant increase in SEVR (*p* = 0.027), and a parallel reduction in SysS after 2 years from bDMARD therapy. In addition, RA patients exhibited a significant decrease in SBP (*p* = 0.023), AIx (*p* = 0.027), and LVET (*p* = 0.02) after 2 years of bDMARD therapy. By contrast, there were no changes in the pulse wave parameters in PsA patients (Figure 1).

**Figure 2:**
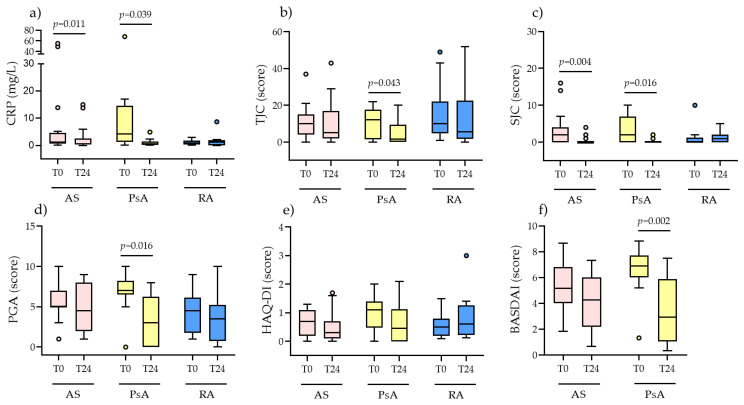
Longitudinal analysis of clinimetric indices. Changes in (**a**) C-reactive protein (CRP); (**b**) tender joint count (TJC); (**c**) swollen joint count (SJC); (**d**) patient global assessment (PGA); (**e**) health assessment questionnaire—disability index (HAQ-DI); (**f**) Bath ankylosing spondylitis disease activity index (BASDAI). Results are reported as median + IQR. Circles are outliers. Comparisons were performed with Wilcoxon matched pairs signed rank test.

Although PsA patients did not show significant changes in pulse wave parameters, they were the group that benefitted most in terms of disease activity. After 24 months of therapy, there was a significant reduction in CRP (*p* = 0.039), TJC (*p* = 0.043), SJC (*p* = 0.016), PGA (*p* = 0.016), and BASDAI (*p* = 0.002), as shown in Figure 2. Similarly, AS patients also showed a significant decrease in CRP levels (*p* = 0.011) and SJC (*p* = 0.004) as shown in Figure 2.

## 4. Discussion

Although the introduction of bDMARDs has greatly improved patient quality of life and modified the natural history of inflammatory arthropathies, the burden of CVD in these patients remains an open issue, being a significant cause of morbidity and mortality [23].

In recent years, aortic stiffness, which refers to the elastic properties of the vessels, has been proposed as a parameter that allows to reclassify CVD risk in several clinical situations [13,14], which seems to be particularly useful in patients without standard CVD risk factors [24,25]. The recommended method to measure aortic stiffness is the non-invasive assessment of carotid–femoral (cf)PWV (i.e., the speed at which the pulse wave travels in the vessel) with applanation tonometry [26]. In addition to cfPWV, applanation tonometry provides also other pulse wave parameters, which give useful information on the cardiovascular system and CVD risk. For instance, AIx is not only a measure of aortic stiffness but it is also related to left ventricular systolic loading, while SEVR is an index of myocardial oxygen supply and demand [27].

In line with the concept that systemic inflammation affects vascular homeostasis, previous studies have shown that patients with rheumatoid arthritis, as well as seronegative spondyloarthritis, exhibited increased arterial stiffness [28,29], which correlated with measures of inflammation and showed a potential reversibility after bDMARD therapy [28].

In our study, we looked at significant associations between pulse wave parameters and general or disease-related characteristics of patients with inflammatory arthropathies. First of all, age was the main clinical variable independently associated with unfavorable pulse wave parameters, namely increased SBPc, higher cfPWV, and LVET. This is consistent with a large body of evidence indicating that the stiffening of vessels, also known as arteriosclerosis, is tightly connected with aging [30]. Second, we found that chronic damage, as assessed by HAQ-DI score, which is one of the most used patient-reported outcomes for the evaluation of the functional status and grade of disability in patients with arthritis [31], was independently and negatively associated with SEVR, which is an established index of myocardial oxygen supply and demand [18]. Since chronic damage is mainly determined by disease activity over time, our results are consistent with previous findings showing a negative association between SEVR and markers of disease activity in patients with RA [32]. Third, OPG levels were also associated with increased cfPWV and SBPc values, but the association that we found at the univariate analysis was not confirmed by linear multivariate analysis. Nevertheless, our finding is consistent with the concept that OPG, which is a member of the TNF receptor superfamily, has emerged as an independent risk factor for CVD [33], possibly by promoting vascular calcifications, leukocyte migration into the vessels, and fibrosis [34,35].

Interestingly, in our study, the PsA group showed a higher baseline cfPWV values as compared to AS and RA. This is in line with the observation that the PsA group had the highest proportion of male subjects, and slightly higher—although not significantly—CRP levels. The highest proportion of male subjects in the PsA group is in line with epidemiological data. Mok et al. showed that in a cohort of patients with AS, PsA and RA, a female predominance was observed in RA patients, whereas PsA had a roughly equal sex incidence [36]. In addition, in the same work, patients with AS were younger compared to RA and PsA [36]. The highest proportion of male subjects in the PsA group can explain the higher baseline cfPWV values given that males have higher global levels of cfPWV [37]. Our data are also in line with the established evidence of a high burden of cardiometabolic diseases in PsA [38] and a worse overall CVD risk profile [36]. In our study, PsA patients showed a tendency towards a higher overall disease activity at baseline, and this may explain the significant improvement over time in the clinimetric indices such as swollen and tender joints (SJC and TJC), as well as the inflammatory markers and overall perception of disease-related disability and symptom intensity (PGA and BASDAI). On the other hand, the higher disease burden, CRP levels, and male subject proportion may have accounted for the lowest impact of bDMARD therapy on arterial elastic properties in this group as compared to the other two.

By contrast, when looking at pulse wave changes after bDMARD therapy in AS and RA patients, we found a significant increase in SEVR, which is an indicator of cardiac perfusion, and a decrease in SysS in the AS group, as well as a significant decrease in SBPc, AIx and LVET in the AR group. These results are in line with the evidence of a reduction in aortic stiffness in patients with RA treated with anti-TNF-α therapy [28], and with the overall reduction in CVD risk in patients treated with bDMARD therapy [39,40]. With regard to the LVET decrease in RA group, it may be argued that the reduction in the ejection time could be associated to a higher risk of heart failure and therefore to an overall worse CVD prognosis. Nevertheless, in the RA group, the decrease in LVET was not associated with a worsening of left ventricular function but it corresponded to its normalization based on the reference ranges reported in the Copenhagen City Heart Study [41].

The main limitations of this study include the small number of patients and the heterogeneity of their clinical and demographic baseline characteristics. Nevertheless, its strengths are the use of validated methods to measure pulse wave parameters and cfPWV, in line with current recommendations [17], the assessment of pulse wave changes over time (and the 24-month follow-up), as well as the inclusion of different types of inflammatory arthropathies.

## 5. Conclusions

It is the current opinion that pulse wave parameters, and especially cfPWV, allow to better define the CVD risk profile in patients with non-standard CV risk factors. Our data confirm that pulse wave parameters are potentially reversible after bDMARD therapy, as they ameliorated in AS and RA patients, who had an improvement in cardiac perfusion and oxygen supply. By contrast, PsA patients, who had the highest male subject proportion and higher baseline cfPWV values, benefited less from bDMARD therapy in terms of vascular changes as compared to AS and RA groups. This is in line with the concept that PsA patients have an overall worse CVD risk profile. Further studies in larger cohorts and with longer follow-up are needed to expand and confirm our findings.

## Figures and Tables

**Table 1:** Baseline patient characteristics.

	AS	PsA	RA	*p*	AS vs. PsA	AS vs. RA	PsA vs. RA
**Number of patients (%)**	16 (44.4%)	10 (27.8%)	10 (27.8%)				
**General characteristics**							
Age; yrs	55 (48–57)	61 (50–74)	71 (65–74)	0.015 *	0.640	0.011 *	0.410
Δage;	0.0 (−1.0–1.3)	1.0 (0.0–4.0)	−1.0 (−2.0–0.0)	0.351			
Sex							
F	14 (87.5%)	6 (60%)	10 (100%)	0.047 *	0.163	0.508	0.087
M	2 (12.5%)	4 (40%)	0 (0%)				
Disease duration; yrs	2.0 (0.8–4.0)	1.0 (0.3–3.0)	4.0 (2.0–6.8)	0.365			
**CVD risk factors**							
DM	2 (12.5%)	2 (20%)	0 (0%)	0.353			
BMI; Kg/m^2^	25.6 (23.0–28.1)	26.0 (25.1–27.5)	26.2 (24.5–27.0)	0.871			
Hypertension	5 (31.25%)	4 (40%)	0 (0%)	0.088			
Smoke	2 (12.5%)	0 (0%)	0 (0%)	0.266			
Lipid profile							
TC; mg/dL	228 (177–234)	230 (204–235)	205 (198–230)	0.866			
TG; mg/dL	82 (70–91)	114 (94–126)	91 (88–129)	0.125			
HDL; mg/dL	64 (54–81)	55 (36–73)	74 (58–77)	0.365			
LDL; mg/dL	136 (108–145)	141 (140–178)	131 (110–134)	0.444			
Statin therapy	3 (18.75%)	2 (20%)	4 (40%)	0.435			
**Clinimetric indices**							
TJC	10 (5–15)	12 (5–17)	10 (6–14)	0.824			
SJC	2 (0–4)	2 (1–7)	0 (0–1)	0.123			
PGA	5 (5–7)	7 (7–8)	5 (2–6)	0.038 *	0.207	1	0.038 *
HAQ-DI	0.7 (0.3–1.1)	1.1 (0.7–1.3)	0.5 (0.2–0.8)	0.317			
**Inflammatory markers**							
CRP; mg/L	1.3 (0.6–3.5)	4.3 (1.7–13.0)	0.9 (0.5–1.5)	0.105			
ESR; mm/h	22 (5–52)	21 (12–33)	30 (10–57)	0.737			
**Cytokines**							
OPG; pg/mL	1257 (1019–1667)	1458 (1174–1853)	1454 (1376–1938)	0.311			
RANKL; pg/mL	9.0 (9.0–104.0)	9.0 (9.0–96.9)	9.3 (9.0–82.8)	0.927			
**Pulse wave parameters**							
cfPWV; m/s	7.9 (7.5–8.7)	9.9 (8.4–11.6)	9.6 (8.3–10.5)	0.048 *	0.075	0.22	1
AIx; %	12.3 (6.4–30.4)	18.5 (−0.9–25.7)	27.0 (23.5–33.5)	0.098			
SEVR; %	104 (91–113)	103 (94–115)	119 (108–141)	0.086			
LVET; ms	306 (290–312)	292 (272–317)	324 (316–329)	0.055			
SysS; mmHg/ms	0.74 (0.53–0.93)	0.60 (0.54–0.81)	0.60 (0.56–0.75)	0.822			
SBPc; mmHg	117 (107–127)	131 (114–132)	131 (126–145)	0.080			
DBPc; mmHg	78 (74–86)	84 (79–88)	81 (80–96)	0.163			

AS, ankylosing spondylitis. PsA, psoriatic arthritis. RA, rheumatoid arthritis. Δage stands for difference between age of birth—vascular age. F, female. M, male. CVD, cardiovascular disease. DM, diabetes mellitus. BMI, body mass index. TC, total cholesterol. TG, triglycerides. HDL, high-density lipoproteins. LDL, low-density lipoproteins. TJC, tender joint count. SJC, swollen joint count. PGA, patient global assessment. HAQ-DI, health assessment questionnaire—disability index. CRP, C-reactive protein. ESR, erythrocyte sedimentation rate. OPG, osteoprotegerin. RANKL, RANK ligand. cfPWV, carotid–femoral pulse wave velocity. AIx, augmentation index. SEVR, subendocardial viability ratio. LVET, left ventricular ejection time. SysS, systolic slope. SBPc, central systolic blood pressure. DBPc, central diastolic blood pressure. Continuous variables are expressed as median (IQR). Continuous variables were compared with Kruskal–Wallis test followed by Dunn’s multiple comparisons test, categorical variables were compared with Fischer test. * *p* < 0.05 was considered statistically significant.

**Table 2:** Associations between patient characteristics and pulse wave parameters at baseline.

	Baseline Pulse Wave Parameters													
	cfPWV		AIx		SEVR		LVET		SysS		SBPc		DBPc	
Continuous Variables	rho	*p*	rho	*p*	rho	*p*	rho	*p*	rho	*p*	rho	*p*	rho	*p*
Age (years)	0.54	0.001 *	0.27	0.12	0.16	0.35	0.38	0.02 *	0.21	0.23	0.47	0.01 *	0.19	0.28
Disease duration (years)	0.031	0.86	0.04	0.81	−0.10	0.57	−0.12	0.49	−0.05	0.76	0.12	0.48	0.16	0.84
BMI (Kg/m^2^)	0.01	0.99	−0.16	0.36	−0.04	0.80	0.05	0.76	−0.16	0.36	−0.22	0.19	−0.22	0.19
TC (mg/dL)	0.01	0.95	0.23	0.25	0.03	0.90	−0.20	0.30	−0.52	0.01 *	0.15	0.47	0.36	0.07
TG (mg/dL)	0.13	0.54	0.13	0.56	−0.09	0.68	−0.21	0.32	0.17	0.42	0.08	0.71	−0.08	0.69
HDL (mg/dL)	−0.07	0.74	0.36	0.09	0.34	0.10	0.09	0.68	−0.19	0.39	0.42	0.04 *	0.57	0.01 *
LDL (mg/dL)	−0.05	0.80	0.06	0.79	−0.13	0.53	−0.26	0.22	−0.50	0.02 *	0.01	0.97	0.21	0.32
CRP (mg/L)	−0.01	0.99	−0.25	0.15	−0.34	0.04 *	0.19	0.25	0.25	0.16	−0.13	0.44	−0.16	0.37
ESR (mm/h)	0.17	0.37	−0.10	0.59	−0.18	0.36	0.21	0.27	0.01	0.97	−0.23	0.23	−0.07	0.73
TJC (*n*)	0.04	0.80	0.29	0.10	−0.05	0.79	−0.13	0.47	−0.09	0.61	−0.04	0.84	−0.04	0.83
SJC (*n*)	0.11	0.52	0.06	0.75	−0.26	0.13	−0.34	0.05 *	0.15	0.41	−0.11	0.53	−0.23	0.19
PGA (score)	0.04	0.83	−0.02	0.91	−0.19	0.27	−0.32	0.06	0.07	0.68	−0.14	0.43	−0.17	0.32
HAQ-DI (score)	0.21	0.24	−0.10	0.57	−0.38	0.03 *	−0.26	0.14	0.05	0.79	−0.01	0.96	−0.12	0.49
OPG (pg/mL)	0.42	0.01 *	0.17	0.32	0.15	0.39	0.27	0.10	0.16	0.36	0.35	0.04 *	0.10	0.57
RANKL (pg/mL)	−0.02	0.89	−0.25	0.14	0.04	0.81	0.06	0.73	−0.16	0.37	−0.03	0.85	−0.01	0.94
**Categorical variables**	**cfPWV**		**AIx**		**SEVR**		**LVET**		**SysS**		**SBPc**		**DBPc**	
Sex (M vs. F)	0.44		0.55		0.14		0.54		0.48		0.72		0.74	
Disease subgroup (PsA, AS, RA)	0.048 *		0.06		0.09		0.055		0.66		0.08		0.10	
Diabetes mellitus (Y vs. N)	0.39		0.90		0.75		0.78		0.29		0.43		0.68	
Hypertension (Y vs. N)	0.07		0.92		0.57		0.53		0.49		-		-	
Statin therapy (Y vs. N)	0.97		0.15		0.08		0.06		0.54		0.25		0.99	

AS, ankylosing spondylitis. PsA, psoriatic arthritis. RA, rheumatoid arthritis. BMI, body mass index. TC, total cholesterol. TG, triglycerides. HDL, high-density lipoproteins. LDL, low-density lipoproteins. TJC, tender joint count. SJC, swollen joint count. PGA, patient global assessment. HAQ-DI, health assessment questionnaire—disability index. CRP, C-reactive protein. ESR, erythrocyte sedimentation rate. OPG, osteoprotegerin. RANKL, RANK ligand. F, female. M, male. Y, yes. N, no. cfPWV, carotid–femoral pulse wave velocity. AIx, augmentation index. SEVR, subendocardial viability ratio. LVET, left ventricular ejection time. SysS, systolic slope. SBPc, central systolic blood pressure. DBPc, central diastolic blood pressure. Associations between two continuous variables were evaluated with the Pearson or the Spearman test based on data distribution. “rho” is the correlation coefficient. Associations with categorical variables were compared with Wilcoxon test or Kruskal–Wallis test followed by Dunn’s multiple comparisons test. * *p* < 0.05 was considered statistically significant.

**Table 3:** Multivariate linear regression for SEVR at baseline.

Predictive Variables	DEPENDENT VARIABLE SEVR		
	β-Estimate	Standard Error	*p* Value
Age	0.50	0.30	0.11
Sex (M)	7.68	10.43	0.47
HAQ-DI	−20.24	7.94	0.016 *

HAQ-DI, health assessment questionnaire—disability index; SEVR subendocardial variability ratio. * *p* < 0.05 is considered statistically significant. Multiple R-squared: 0.25, Adjusted R-squared: 0.17, *p*-value 0.04.

## Data Availability

Raw data that support the findings of this study are available from the corresponding author, upon reasonable request.
